# Unsteady nanofluid flow over a cone featuring mixed convection and variable viscosity

**DOI:** 10.1016/j.heliyon.2023.e16393

**Published:** 2023-05-30

**Authors:** Zubair Mustafa, T. Javed, T. Hayat, A. Alsaedi

**Affiliations:** a Department of Mathematics and Statistics, International Islamic University, Islamabad, 44000, Pakistan; b Department of Mathematics, Quaid-i-Azam University, Islamabad, 44000, Pakistan; c Nonlinear Analysis and Applied Mathematics (NAAM) Research Group, Faculty of Science, King Abdulaziz University, Jeddah, 21589, Saudi Arabia

**Keywords:** Nanofluids, Mixed convection, Viscous dissipation, Variable viscosity, Rotating cone, Homotopy analysis method

## Abstract

This article addresses unsteady nanofluid flow over a cone with MHD and mixed convection effects. Effects of variable viscosity and viscous dissipation are also considered. The resulting system of equations is tackled through the Homotopy Analysis Method (HAM). The impact of different influential variables on skin friction coefficient, heat and mass flux are discovered through numerical tables and graphs. It is noted that the surface drag force in x and y directions increases against the buoyancy force parameter. Also, it is observed that the tangential and azimuthal velocity decrease against the variable viscosity parameter. Furthermore, the temperature of fluid is observed to decay against the unsteady parameter but it increases against the Eckert number.

## Nomenclature

$\alpha_{1}$: angular velocity ratio
$D_{B}$: Brownian diffusion
$N_{b}$: Brownian motion parameter
$\lambda_{1}$: buoyancy parameter
$N_{1}$: buoyancy forces ratio parameter
c: concentration
ρ: density
μ: dynamic viscosity
Ec: Eckert number
σ: electrical conductivity
$\left(Gr_{1},Gr_{2}\right)$: Grashof numbers
${\left(\rho c\right)}_{f}$: heat capacity (fluid)
${\left(\rho c\right)}_{p}$: heat capacity (nano-particles)
ν: kinematic viscosity
M: magnetic parameter
Pr: Prandtl number
Sc: Schmidt number
$\alpha^{*}$: semi vertical angle (cone)
K: thermal diffusivity
$D_{T}$: Thermophoresis diffusion
$N_{t}$: Thermophoresis parameter
T: Temperature
s: unsteady parameter
$\beta^{*}$: volumetric coefficient for concentration
$\beta_{1}$: volumetric coefficient for temperature

## 1 Introduction

The study of mixed convection flows due to numerous engineering and industrial applications, including fans, furnaces, nuclear reactors, solar powered, automobile, heat exchangers, electronic devices, jets, biological productions, evaporators, chemical processing equipment and geothermal reservoirs has attracted the attention of scientists. In addition, several scientists and researchers have recently investigated mixed convection in rotating cone by various devices, such as spin-stabilized missiles, geothermal reservoirs, heat dissipation and nuclear waste disposal. Himasekhar et al. [1]investigated the consequences of mixed convection in rotating cone flow. Kumari et al. [2] solved the above problem for unsteady flow. Anilkumar and Roy [3] found that both tangential and circumferential velocities have decreased for magnetic parameter. Hayat et al. [4] discussed mixed convective flow over a non-linearly stretching surface. Nadeem and Saleem [5] used the Homotopy analysis method (HAM) for their analysis. Some articles about mixed convection flow are highlighted in Refs. [[6], [7], [8], [9], [10]].

Many researchers have studied the Magnetohydrodynamics (MHD) and variable viscosity effects due to its numerous contributions in technology and engineering. Shit and Majee [11] examined the unsteady flow with a magnetic field and variable viscosity depending upon high temperature. Makinde et al. [12] studied the incompressible fluid with chemical reactions, variable viscosity, heat transfer and thermophoresis. They solved nonlinear differential equations by applying a Runge-kutta integration technique. Khan et al. [13] investigated the result of variable viscosity based on nanofluids over a stretching surface. They showed that when thermophoresis and Brownian parameters are increased, the concentration profile decreases. Umavathi et al. [14] analyzed that the thermal conductivity and variable viscosity increased with temperature. Manjunatha and Gireesha [15] showed that when the dusty fluid is decreased, the variable viscosity and temperature profile increase. Some articles about this topic are seen in Refs. [[16], [17], [18], [19], [20], [21]].

The dynamics of the heat transfer phenomenon on nanoparticles has captivated the focus of scientists. Nanofluids have many applications in compressors, temperature, fins, hybrid-powered machines, refrigerators, nano-cryosurgery and sensing. Choi [22] introduced the word nanofluid. Xuan and Li [23] anticipated a procedure to extend nanomaterials with nanophase powder. Khanafer et al. [24] studied a series of volume fractions and Grashof numbers. Relevant research work is given in Refs. [[25], [26], [27], [28], [29], [30], [31], [32], [33], [34], [35], [36], [37], [38], [39], [40], [41], [42], [43], [44], [45], [46], [47], [48], [49]].

The above studies testify that no attempt has been made to investigate the analysis of unsteady mixed convection and Magnetohydrodynamics flow by a rotating cone. Therefore, our main effort is to attempt the effects of variable viscosity, viscous dissipation and nanofluids on the cone. The resulting equations are solved by HAM. In addition, we have shown graphs and tables for various parameters in our study.

## 2 Modelling

**Fig. 1:**
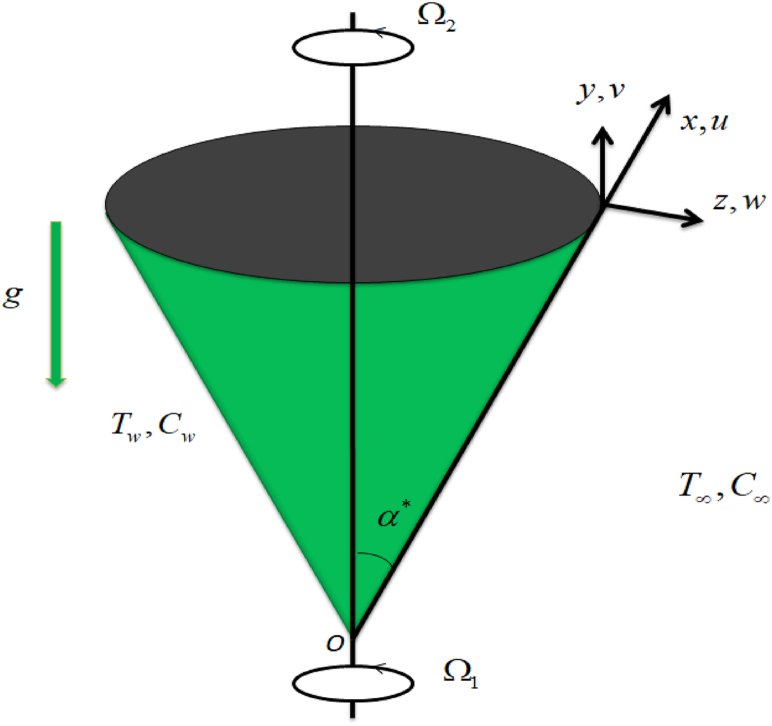
Problem Sketch.

Consider unsteady MHD flow due to the rotation of a cone. Rectangular curvilinear coordinates are assumed. Let (u,v) and (w) are velocities in (x,y) and (z) directions. Here (x) denotes tangential, (y) circumferential and (z) normal directions to the cone and $v_{e}=\left(\frac{\sin\;\alpha^{*}\Omega_{2}x}{1-st^{*}}\right)$ is the free-stream velocity. Acceleration due to gravity (g) acts downwards. The wall concentration is $\left(C_{w}\right)$ and wall temperature is $\left(T_{w}\right)$ and $\Omega =\Omega_{1}+\Omega_{2}$ represent composite angular velocity, $\Omega_{1}$ free stream fluid and $\Omega_{2}$ is the angular velocity of the cone respectively. $\left(T_{\infty },C_{\infty }\right)$ are denoted by ambient temperature and ambient concentration respectively. A small magnetic Reynolds number is taken for the negligible impact of the induced magnetic field. Further, our interest is to carry out the analysis of viscous dissipation and viscosity effects. Thermophoresis and Brownian motion are analyzed. A physical model can be seen in Fig. 1.

Consider variable viscosity is:

(1) $$\mu =\mu_{0}e^{-\xi_{1}\left(T-T_{\infty }\right)}\text{,}$$

(2) $$\mu =\mu_{0}\left(1-A\theta \right)\text{,}$$

where $A=\xi_{1}\left(T_{w}-T_{\infty }\right)$ and $\mu_{0}$ is viscosity of fluid.

The governing equations are as follows (see Refs. [3,5,9,10]).

(3) $$\frac{\partial \left(xu\right)}{\partial x}+\frac{\partial \left(xw\right)}{\partial z}=0\text{,}$$

(4) $$\frac{\partial u}{\partial t}+u\frac{\partial u}{\partial x}+w\frac{\partial u}{\partial z}-\frac{v^{2}}{x}=\frac{1}{\rho }\frac{\partial }{\partial z}\left(\mu \frac{\partial u}{\partial z}\right)-\frac{v_{e}^{2}}{x}+g\beta^{*}\;\cos\;\alpha^{*}\left(C-C_{\infty }\right)+g\beta_{1}\;\cos\;\alpha^{*}\left(T-T_{\infty }\right)-\frac{\sigma }{\rho }B^{2}u\text{,}\}$$

(5) $$\frac{\partial v}{\partial t}+u\frac{\partial v}{\partial x}+\frac{uv}{x}+w\frac{\partial v}{\partial z}=\frac{\partial }{\partial z}\left(\mu \frac{\partial v}{\partial z}\right)\frac{1}{\rho }+\frac{\partial v_{e}}{\partial t}-\frac{\sigma }{\rho }B^{2}\left(v-v_{e}\right)\text{,}$$

(6) $$\begin{array}{l} {\left(\rho c\right)}_{f}\frac{\partial T}{\partial t}+{\left(\rho c\right)}_{f}u\frac{\partial T}{\partial x}+{\left(\rho c\right)}_{f}w\frac{\partial T}{\partial z}=k\frac{\partial^{2}T}{\partial z^{2}}+{\left(\rho c\right)}_{p}\;\left[\frac{D_{T}}{T_{\infty }}{\left(\frac{\partial T}{\partial z}\right)}^{2}+D_{B}\frac{\partial T}{\partial z}\frac{\partial c}{\partial z}\right]+\mu \left[{\left(\frac{\partial u}{\partial z}\right)}^{2}+{\left(\frac{\partial v}{\partial z}\right)}^{2}\right],\} \end{array}$$

(7) $$\frac{\partial c}{\partial t}+w\frac{\partial c}{\partial z}+u\frac{\partial c}{\partial x}=\frac{D_{T}}{T_{\infty }}\frac{\partial^{2}T}{\partial z^{2}}+D_{B}\frac{\partial^{2}c}{\partial z^{2}}\text{,}$$

with

(8) $$\begin{array}{l} w\left(t,x,0\right)=0=u\left(t,x,0\right),v\left(t,x,0\right)=\Omega_{1}x\;\sin\;\alpha^{*}{\left(1-st^{*}\right)}^{-1},C\left(t,x,\infty \right)=C_{\infty }\text{.}\\ u\left(t,x,\infty \right)=0,T\left(t,x,0\right)=T_{w},v\left(t,x,\infty \right)=\Omega_{2}x\;\sin\;\alpha^{*}{\left(1-st^{*}\right)}^{-1},C\left(t,x,0\right)=C_{w},T\left(t,x,\infty \right)=T_{\infty }\text{.} \end{array}\}$$

Transformations and dimensionless variables are:

$$u=-\frac{1}{2\left(1-st^{*}\right)}\Omega x\;\sin\;\alpha^{*}f^{\prime }\left(\eta \right),v=\frac{\Omega x\;\sin\;\alpha^{*}g\left(\eta \right)}{\left(1-st^{*}\right)}\text{,}$$

$$Gr_{2}=\frac{{L_{1}}^{3}}{v^{2}}g\beta^{*}\;\cos\;\alpha^{*}\left(C_{0}-C_{\infty }\right),\eta ={\left(\frac{\sin\;\alpha^{*}\Omega }{v}\right)}^{1/2}\frac{z}{{\left(1-st^{*}\right)}^{1/2}}\text{,}$$

$$t^{*}=t\left(\Omega \;\sin\;\alpha^{*}\right),w=\frac{f\left(\eta \right)}{{\left(1-st^{*}\right)}^{1/2}}{\left(v\Omega \;\sin\;\alpha^{*}\right)}^{1/2},\lambda_{2}=\frac{Gr_{2}}{{Re}_{L_{1}}^{2}}\text{,}$$

$$T_{w}-T_{\infty }=\left(T_{0}-T_{\infty }\right)\left(\frac{x}{L_{1}}\right)\frac{1}{{\left(1-st^{*}\right)}^{2}},C-C_{\infty }=\varphi \left(\eta \right)\left(C_{w}-C_{\infty }\right)\text{,}$$

$$\left(C_{w}-C_{\infty }\right)=\left(\frac{x}{L_{1}}\right)\left(C_{0}-C_{\infty }\right)\frac{1}{{\left(1-st^{*}\right)}^{2}},T-T_{\infty }=\theta \left(\eta \right)\left(T_{w}-T_{\infty }\right)\text{,}$$

$$M=\frac{\left(1-st^{*}\right)}{\left(\Omega \;\sin\;\alpha^{*}\right)}\frac{\sigma B_{0}^{2}}{\rho },N_{1}=\frac{\lambda_{2}}{\lambda_{1}},Sc=\frac{\nu }{D_{B}},\mathrm{Pr}=\frac{v}{\alpha },\alpha_{1}=\frac{\Omega_{1}}{\Omega }\text{,}$$

$$Ec=\frac{L^{2}{\left(\Omega \;\sin\;\alpha^{*}\right)}^{2}}{c_{p}\left(T_{0}-T_{\infty }\right)},N_{b}=\frac{{\left(\rho c\right)}_{p}D_{B}\left(C_{w}-C_{\infty }\right)}{v{\left(\rho c\right)}_{f}},\xi =\frac{x}{L},\lambda_{1}=\frac{Gr_{1}}{{Re}_{L_{1}}^{2}}\text{,}$$

(9) $$N_{t}=\frac{{\left(\rho c\right)}_{p}D_{T}\left(T_{w}-T_{\infty }\right)}{v{\left(\rho c\right)}_{f}T_{\infty }},Gr_{1}=\frac{{L_{1}}^{3}}{v^{2}}g\beta_{1}\;\cos\;\alpha^{*}\left(T_{0}-T_{\infty }\right),{Re}_{L_{1}}=\frac{{L_{1}}^{2}}{v}\Omega \;\sin\;\alpha^{*}\text{.}$$

After using these dimensionless variables and considering $\left(\frac{\partial }{\partial \xi }\right)$ and its derivatives equal to zero, we get,

$$\left(1-A\theta \right)f^{\prime \prime \prime }-A\theta^{\prime }f^{\prime \prime }-ff^{\prime \prime }-s\left(f^{\prime }+\frac{\eta f^{\prime \prime }}{2}\right)-2\left(g^{2}-{\left(1-\alpha_{1}\right)}^{2}\right)+\frac{{f^{\prime }}^{2}}{2}-2\lambda_{1}\left(\theta +N_{1}\varphi \right)-Mf^{\prime }=0\text{,}$$

(11) $$\left(1-A\theta \right)g^{\prime \prime }-A\theta^{\prime }g^{\prime }-\left(fg^{\prime }-gf^{\prime }\right)-M\left(g-1+\alpha_{1}\right)+s\left(1-g-\alpha_{1}-\frac{\eta g^{\prime }}{2}\right)=0\text{,}$$

(12) $$\left(\frac{1}{\mathrm{Pr}}\right)\;\theta^{\prime \prime }-s\left(2\theta +\frac{1}{2}\eta \theta^{\prime }\right)-\left(f\theta^{\prime }-f^{\prime }\frac{\theta }{2}\right)+N_{t}{\theta^{\prime }}^{2}+N_{b}\theta^{\prime }\varphi^{\prime }+\left(1-A\theta \right)Ec\xi \left(\frac{1}{4}{\left(f^{\prime \prime }\right)}^{2}+{\left(g^{\prime }\right)}^{2}\right)=0\text{,}$$

(13) $$\frac{1}{Sc}\varphi^{\prime \prime }-f\varphi^{\prime }+f^{\prime }\frac{\varphi }{2}-s\left(\frac{1}{2}\eta \varphi^{\prime }+2\varphi \right)+\frac{N_{t}}{N_{b}}\theta^{\prime \prime }=0\text{,}$$

$$f\left(0\right)=0=f^{\prime }\left(0\right),g\left(0\right)=\alpha_{1},f^{\prime }\left(\infty \right)=0,g\left(\infty \right)=1-\alpha_{1}\text{,}$$

(14) θ(0)=φ(0)=1,θ(∞)=φ(∞)=0.

The skin friction in x and y direction are

(15) $$C_{fx}=\frac{{\left[2\mu \left(\frac{\partial u}{\partial z}\right)\right]}_{z=0}}{\rho {\left[\Omega x\;\sin\;\alpha^{*}{\left(1-st^{*}\right)}^{-1}\right]}^{2}},C_{fy}=-\frac{{\left[2\mu \left(\frac{\partial v}{\partial z}\right)\right]}_{z=0}}{\rho {\left[\Omega x\;\sin\;\alpha^{*}{\left(1-st^{*}\right)}^{-1}\right]}^{2}}\text{,}$$

or

(16) $$\xi C_{fx}{Re}_{L}^{1/2}={\left[-\left(1-A\theta \right)f^{\prime \prime }\right]}_{\eta =0},\xi C_{fy}{Re}_{L}^{1/2}={\left[-\left(1-A\theta \right)g^{\prime }\right]}_{\eta =0}\text{.}$$

Where ${Re}_{L}=\frac{\Omega L^{2}\;\sin\;\alpha^{*}}{v\left(1-st^{*}\right)}$ is the Reynolds number.

The Sherwood and Nusselt numbers are

(17) $$\xi Sh{Re}_{L}^{-1/2}=-\varphi^{\prime }\left(0\right),\xi Nu{Re}_{L}^{-1/2}=-\theta^{\prime }\left(0\right)\text{.}$$

## 3 Solution methodology

**Fig. 2:**
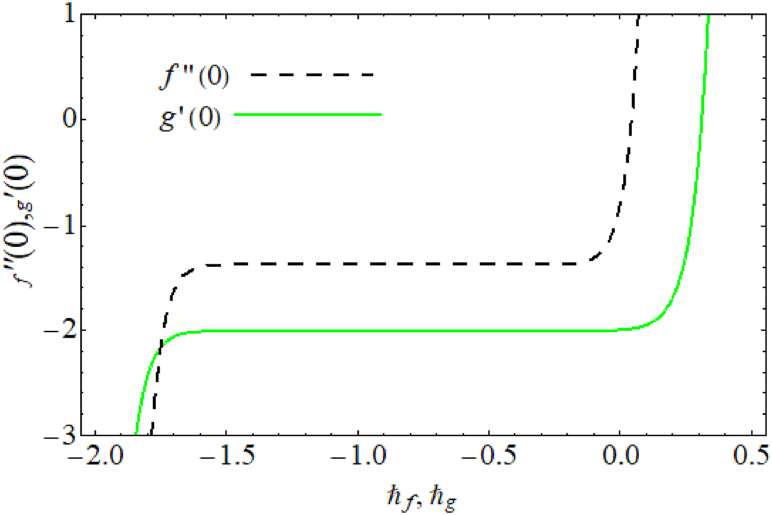
ℏ− curves for velocity.

**Fig. 3:**
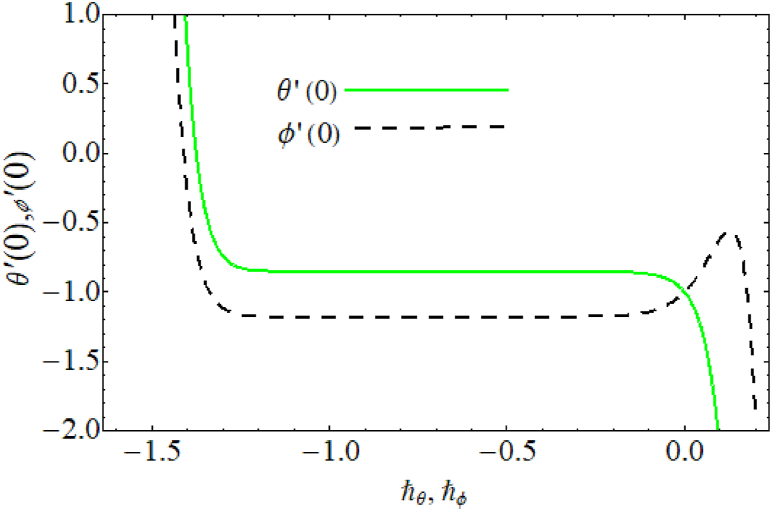
ℏ− curves for temperature and concentration.

Equations (10)−(14) are solved by HAM. HAM is valid for large as well as small values of parameters and the method also guarantees convergent solutions. Equations (18)−(25) are defined initial guesses and linear operators. The dependence of convergence of solutions is on (auxiliary variables) $\hbar_{f},\hbar_{g},\hbar_{\theta },\hbar_{\varphi }$. The ranges of $\hbar_{f}\text{,}$ $\hbar_{g},\hbar_{\theta }$ and $\hbar_{\varphi }$ is −1.5 ≤ $\hbar_{f}$ ≤ −0.4 , −1.5 ≤ $\hbar_{g}$ ≤ −0.3 , −1.2 ≤ $\hbar_{\theta }$ ≤ −0.3 and −1.1 ≤ $\hbar_{\varphi }$ ≤ −0.4 are seen in Fig. 2, Fig. 3. Table 1 shows the solutions convergence.

(18) $$f_{0}\left(\eta \right)=0\text{,}$$

(19) $$g_{0}\left(\eta \right)=\exp\left(-\eta \right)\left(2\alpha_{1}-1\right)+\left(1-\alpha_{1}\right)\text{,}$$

(20) $$\varphi_{0}\left(\eta \right)=\exp\left(-\eta \right)\text{,}$$

(21) $$\theta_{0}\left(\eta \right)=\exp\left(-\eta \right)\text{,}$$

(22) $$L_{f}=-f^{\prime }+f^{\prime \prime \prime }\text{,}$$

(23) $$L_{g}=g^{\prime \prime }-g\text{,}$$

(24) $$L_{\theta }=\theta^{\prime \prime }-\theta \text{,}$$

(25) $$L_{\varphi }=\varphi^{\prime \prime }-\varphi \text{.}$$

**Table 1:** Convergence of solutions.

Order of convergence	-$f^{\prime \prime }\left(0\right)$	-$g^{\prime }\left(0\right)$	-$\theta^{\prime }\left(0\right)$	-$\varphi^{\prime }\left(0\right)$
**1**	0.5823	1.2342	1.5928	0.6340
**10**	0.6719	1.3427	1.5872	0.5948
**14**	0.6923	1.3846	1.5673	0.5837
**20**	0.6923	1.3846	1.5673	0.5837
**25**	0.6923	1.3846	1.5673	0.5837
**30**	0.6923	1.3846	1.5673	0.5837

## 4 Graphs and tables

.

## 5 Discussion

Here we examine outcomes of (s) , (M) , $\left(\lambda_{1}\right)$ , $\left(N_{1}\right)$ , (Sc) , $\left(N_{b}\right)$ , $\left(\alpha_{1}\right)$ , (Pr), $\left(N_{t}\right)$ , (Ec) and (A) on temperature, velocity and concentration. The heat transfer, mass flux and skin friction numerical values are presented in the tables.

**Fig. 4:**
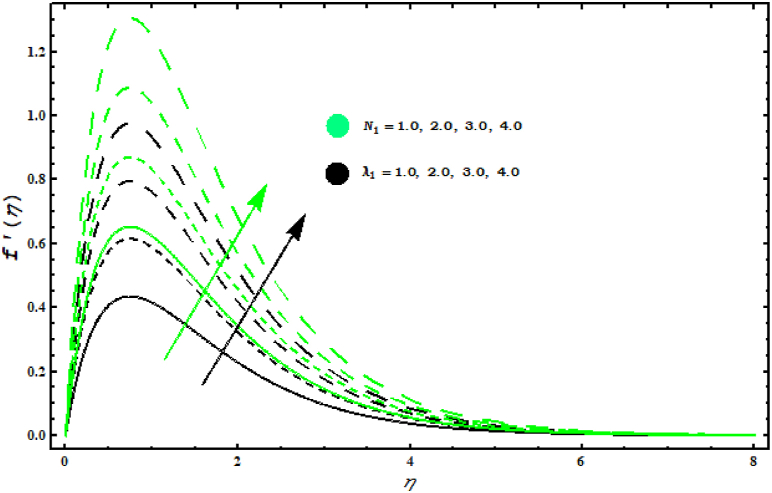
Impact of $N_{1}$ and $\lambda_{1}$ on $f^{\prime }\left(\eta \right)\text{.}$.

**Fig. 5:**
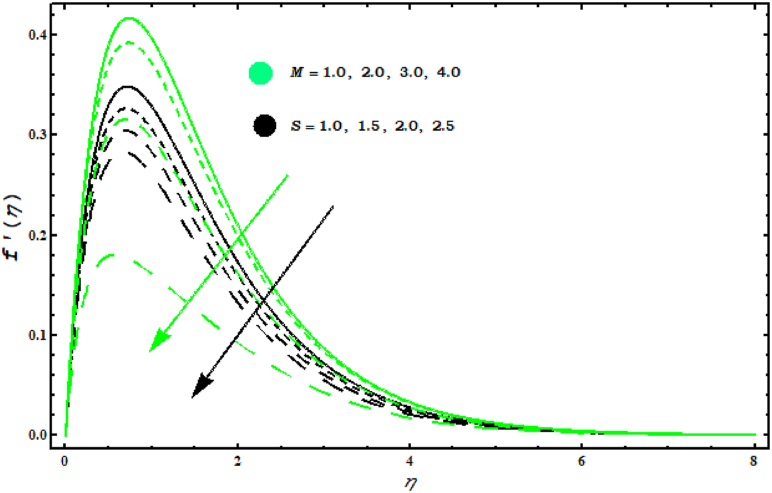
Impact of M and S on $f^{\prime }\left(\eta \right)\text{.}$.

**Fig. 6:**
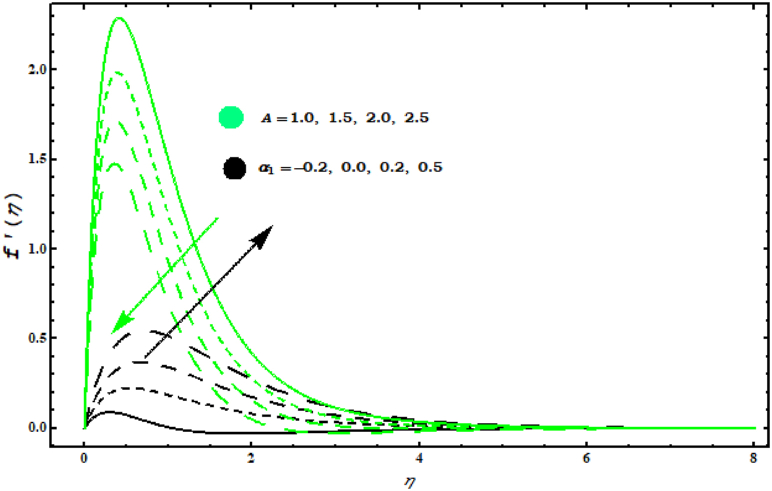
Impact of A and $\alpha_{1}$ on $f^{\prime }\left(\eta \right)\text{.}$.

**Fig. 7:**
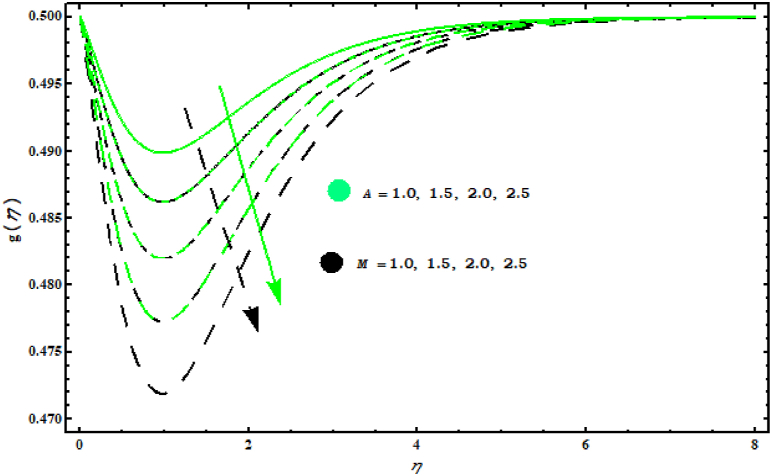
Impact of A and M on g(η)..

Fig. 4, Fig. 5, Fig. 6, Fig. 7study the velocity graph for different parameters. Figure 4 shows the variation in tangential velocity $f^{\prime }\left(\eta \right)$ for $(N_{1}$, $\lambda_{1}$). Increasing behaviour in $f^{\prime }\left(\eta \right)$ is evidenced by increasing values of $(N_{1}$, $\lambda_{1}$). Thermal gradient growth is reduced by physically higher buoyancy parameters. The viscosity and consequently the velocity of the fluid increases. Fig. 5 is devoted to the interpretation of the behaviour of (M, S) in $f^{\prime }\left(\eta \right)\text{.}$ Fig. 5 shows that $f^{\prime }\left(\eta \right)$ decreases for increasing values of (M). Also, it is seen that velocity in tangential direction gears down against unsteady parameter. This random movement offers more resistance and the flow of elements is reduced. Fig. 6shows the behaviour of $f^{\prime }\left(\eta \right)$ for (A, $\alpha_{1}$). It is observed that $f^{\prime }\left(\eta \right)$ increases against $\left(\alpha_{1}\right)$ while it is the opposite of behaviour against parameter (A). Physically, it is valid because by increasing (A) thickness of fluid is strengthened which deteriorates the velocity. When $\alpha_{1}=0.5$ the rotation of fluid and cone are similar. For $\alpha_{1}>0.5$ velocity increases in tangential direction. Fig. 7 demonstrates the impact of (A , M) on circumferential velocity g(η). Clearly, g(η) is reduced for (A , M).

**Fig. 8:**
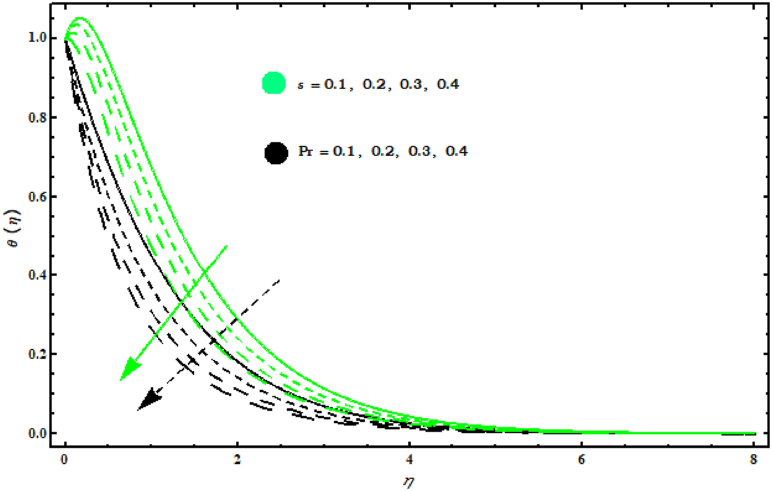
Impact of s and Pr on θ(η)..

**Fig. 9:**
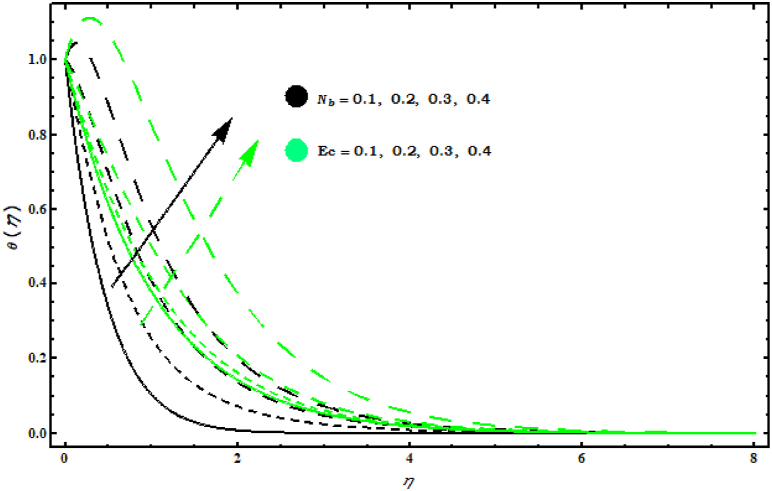
Impact of $N_{b}$ and Ec on θ(η)..

**Fig. 10:**
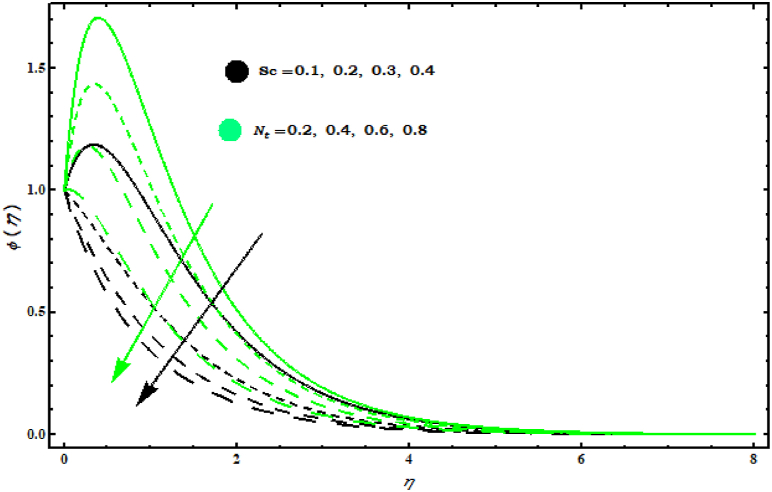
Impact of Sc and $N_{t}$ on φ(η)..

8−10 display the temperature and concentration fields when several parameters are varied. Effects of (s, Pr) on θ(η) are displayed in Fig. 8 . It is observed that θ(η) decreases by increasing (s, Pr). This graph shows that increasing (Pr) decreases the temperature profile because (Pr) is the ratio of viscosity (ν) to thermal diffusivity (α) and increasing (Pr) decreases thermal diffusivity thus it reduces heat spread from the cone surface. Fig. 9 shows that θ(η) increases with respect to ($N_{b}$, Ec). Physically, an increase in magnitude $\left(N_{b}\right)$ results in effective movement of fluid particles which tends to increase thermal conductivity of fluid and as a consequence temperature of fluid increases. As (Ec) represents the fraction of kinetic energy for the enthalpy difference from heat. Hence an increase in (Ec) causes an increase in kinetic energy. Also, it is well known that temperature is a measure of the average kinetic energy of the molecules of a fluid. Thus, the temperature of the fluid increases with the increase of (Ec). Fig. 10is displayed the effects of ($N_{t}$, Sc) on φ(η). It is seen that both ($N_{t}$ , Sc) give same behaviour on φ(η)..

**Fig. 11:**
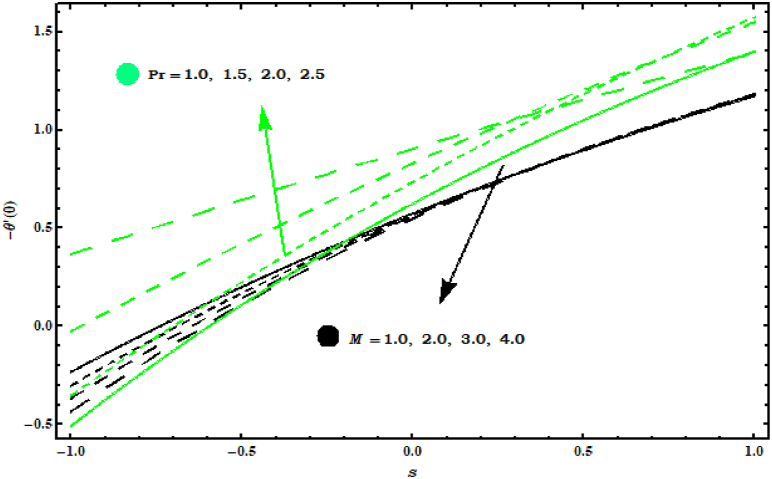
Impact of Pr and M on $-\theta^{\prime }\left(0\right)\text{.}$.

**Fig. 12:**
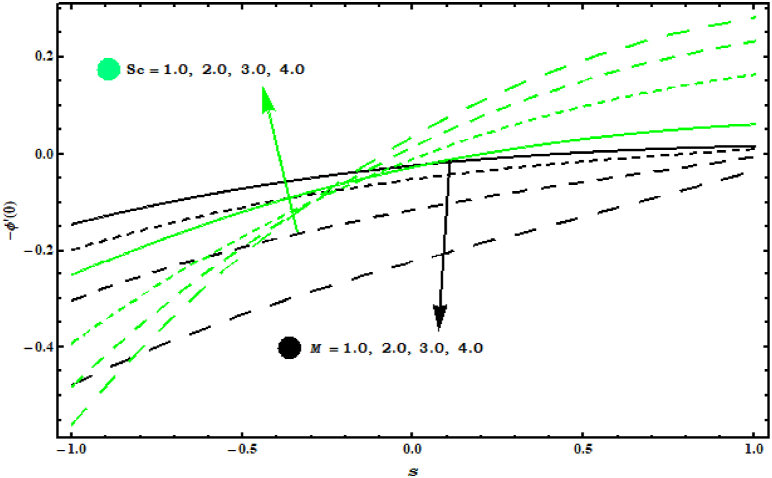
Impact of Sc and M on $-\varphi^{\prime }\left(0\right)\text{.}$.

Fig. 11and 12show the Nusselt numbers $-\theta^{\prime }\left(0\right)$ and Sherwood numbers $-\varphi^{\prime }\left(0\right)$ graph for various parameters. $-\theta^{\prime }\left(0\right)$ is increasing behaviour for (Pr) and reducing behaviour is noticed for (M) (see Figure 11). In Figure 12 enhancement in $-\varphi^{\prime }\left(0\right)$ is observed for (Sc) and reducing behaviour is seen against (M) .

**Fig. 13:**
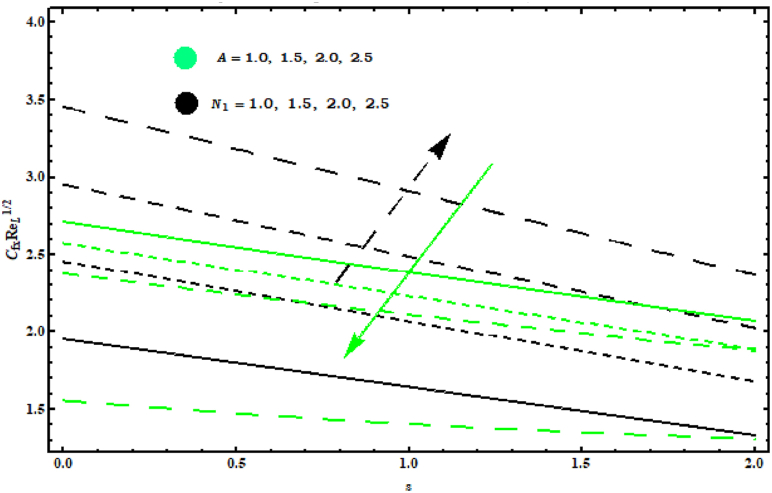
Impact of A and $N_{1}$ on $C_{fx}{Re}_{L}^{1/2}\text{.}$.

**Fig. 14:**
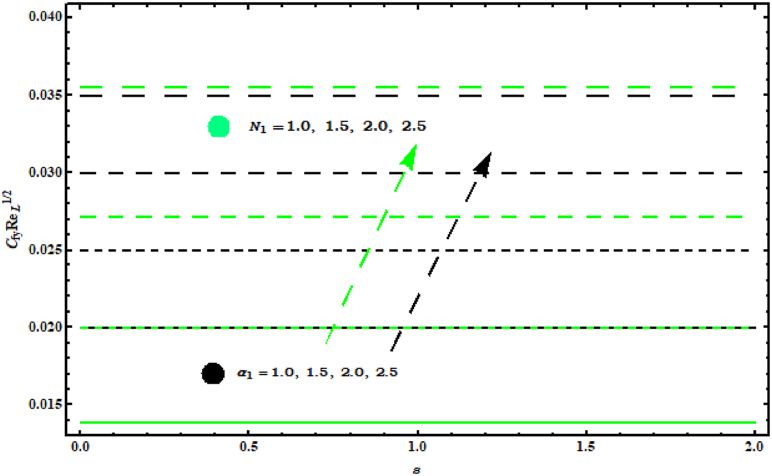
Impact of $N_{1}$ and $\alpha_{1}$ on $C_{fy}\;{Re}_{L}^{1/2}\text{.}$.

The effects of $\left(N_{1}\right)$, (A) and $\left(\alpha_{1}\right)$ on the $\left(C_{fx}{Re}_{L}^{1/2}\right)$ and $\left(C_{fy}{Re}_{L}^{1/2}\right)$ are displayed in Figs. 13 and 14. Impacts of (A, $N_{1}$) on $\left(C_{fx}{Re}_{L}^{1/2}\right)$ are designed in Fig. 13 . In this figure $\left(C_{fx}{Re}_{L}^{1/2}\right)$ increasing for $\left(N_{1}\right)$ and decreasing for (A) . In fact $\left(N_{1}\right)$ shows buoyancy forces ratio twisted towards concentration and temperature changes. Hence for higher $\left(N_{1}\right)$ wall temperature enhances and surface drag force enlarges. Behaviour of $\left(C_{fy}{Re}_{L}^{1/2}\right)$ for ($N_{1}$ , $\alpha_{1}$) is revealed in Fig. 14 . Both parameter ($N_{1}$ , $\alpha_{1}$) causes increase in $\left(C_{fy}{Re}_{L}^{1/2}\right)\text{.}$.

Table 2display numerical values for the skin friction along tangential direction $\left(C_{fx}{Re}_{L}^{1/2}\right)$ and circumferential direction $\left(C_{fy}{Re}_{L}^{1/2}\right)$ for wide range of (A) , (s), $\left(\alpha_{1}\right)$ and $\left(N_{1}\right)$ . It is evaluated from computed numerical values that friction factor along tangential and circumferential direction increase for $\left(N_{1}\right)$. Table 3 shows heat transfer enlarges for increasing (Pr) while opposite pattern is seen for Sherwood number. Comparison of the present values of temperature gradient with previous studies [9,10] is given in Table 4.

**Table 2:** Surface drag force values of $A,s,\alpha_{1},N_{1}$.

A	s	$\alpha_{1}$	$N_{1}$	$C_{fx}\;{Re}_{L}^{1/2}$	$C_{fy}\;{Re}_{L}^{1/2}$
**0.2**				0.6875	0.5813
**0.4**				0.5473	0.6720
**0.6**				0.4538	0.7528
	0.2			1.6752	0.1845
	0.3			1.5437	0.2237
	0.4			1.3849	0.3176
		0.6		0.7445	0.5327
		0.7		0.6348	0.6254
		0.8		0.5863	0.7253
			1.0	1.3457	1.2732
			1.5	1.4268	1.3465
			2.0	1.5728	1.4579

**Table 3:** Heat and mass transfer for Pr,A,Ec,Sc..

Pr	A	Ec	Sc	$Nu\;{Re}_{L}^{-1/2}$	$Sh\;{Re}_{L}^{-1/2}$
**0.1**				0.5782	1.0752
**0.2**				0.6320	1.0627
**0.3**				0.8214	1.0573
	1.0			0.8762	0.5312
	2.0			0.8462	0.5635
	3.0			0.8139	0.5972
		0.3		0.4735	0.5647
		0.4		0.4173	0.6381
		0.5		0.3521	0.7452
			0.1	0.7823	0.6251
			0.3	0.6952	0.7135
			0.5	0.5364	0.7738

**Table 4:** Comparison of temperature gradient with Khan et al. [9] and Chamkha and Rashad [10].

Pr	$\lambda_{1}$	Present values	Khan et al. [9]	Chamkha and Rashad [10]
**0.7**	0	0.4299	0.4299	0.4299
	1	0.6122	0.6120	0.6120
	10	1.3992	1.3992	1.0097
**10**	0	1.4112	1.4113	1.4110
	1	1.5661	1.5663	1.5662
	10	2.3581	2.3582	2.3580

## 6 Conclusions

This work deals with the unsteady nanofluid flow over a cone with the effects of variable viscosity, mixed convection and MHD. Dissipation, Brownian and thermophoresis effects are also considered in energy equation. The important points are listed below.

F0B7 $f^{\prime }\left(\eta \right)$ is decreasing for (M) and (s)..
F0B7 Velocity components $f^{\prime }\left(\eta \right)$ and g(η) have same behaviour for (A).
F0B7 $f^{\prime }\left(\eta \right)$ boosts up for $\left(\lambda_{1}\right)$ and $\left(N_{1}\right)$.
F0B7 θ(η) is decreased with increasing (Pr) and (s).
F0B7 Concentration field φ(η) against (Sc) decreases.
F0B7 $\left(C_{fx}{Re}_{L}^{1/2}\right)$ and $\left(C_{fy}{Re}_{L}^{1/2}\right)$ have same behaviour against $\left(N_{1}\right)\text{.}$.
F0B7 $\left(Nu{Re}_{L}^{-1/2}\right)$ and $\left(Sh{Re}_{L}^{-1/2}\right)$ decrease against (M).

In future this work can be extended for non-Newtonian fluids. Furthermore the addition of hybrid nanofluid, thermal radiation, chemical reaction and conductivity may play a significant role in this direction.

## Author contribution statement

**Zubair Mustafa:** Performed the experiments, wrote the paper.

**T. Javed:** Conceived and designed the experiments; Analyzed and interpreted the data.

**T. Hayat:** Contributed reagents, materials, analysis tools or data.

**A. Alsaedi:** Analyzed and interpreted the data.

## Data availability statement

Data will be made available on request.

## Declaration of competing interest

The authors declare that they have no conflict of interest with anybody exist.
